# A graphdiyne-enabled TiO_2_/g-C_3_N_4_ bio-heterojunction for visible-light antibacterial photocatalysis and infected wound healing

**DOI:** 10.1016/j.mtbio.2026.103765

**Published:** 2026-10-20

**Authors:** Chongqing Yu, Zujiang Liu, Jin Ye, Ruixin Zhang, Min Ren, Fulin Deng, Anning Zhao, Ali Abou-Hassan, Yichun Wang, Yao Zhang, Valentim A.R. Barão, Liqun Xu, Jie Xu, Xi Rao

**Affiliations:** a BRICS Joint Laboratory on Biomedical Materials, School of Materials and Energy, Southwest University, Chongqing, 400715, China; b College of Stomatology, Chongqing Medical University; Chongqing Key Laboratory of Oral Diseases; Chongqing Municipal Key Laboratory of Oral Biomedical Engineering of Higher Education; Chongqing Municipal Health Commission Key Laboratory of Oral Biomedical Engineering, Chongqing, 401147, China; c PHysicochimie des Électrolytes et Nanosystèmes InterfaciauX (PHENIX), Sorbonne Université, CNRS, 75005, Paris, France; d Institut Universitaire de France (IUF), 75231, Paris, France; e Department of Prosthodontics and Periodontology, Piracicaba Dental School, University of Campinas (UNICAMP), Piracicaba, São Paulo, Brazil

**Keywords:** Graphdiyne, Bio-heterojunction, TiO_2_/g-C_3_N_4_, Visible-light antibacterial photocatalysis, Infected wound healing, *MRSA*

## Abstract

Visible-light-driven antibacterial photocatalysis holds great promise for infected wound management; however, efficient and biologically compatible heterointerfaces remain limited. Here, we constructed a graphdiyne-enabled TiO_2_/g-C_3_N_4_ based bio-heterojunction (TCN/GDY) by growing graphdiyne (GDY) onto a TiO_2_/g-C_3_N_4_ scaffold, which couples enhanced visible-light harvesting with antibacterial wound therapy. Structural characterization, photoelectrochemical measurements, valence band analysis, *in situ* XPS, electron paramagnetic resonance, and density functional theory support interfacial electronic coupling, an enhanced photoresponse, and reactive oxygen species generation. These observations are consistent with a proposed dual S-scheme like mechanism. Under 420 nm LED irradiation, TCN/GDY achieved >99% elimination of *Escherichia coli*, 99.40 ± 0.34% elimination of *Staphylococcus aureus*, and 98.65 ± 0.74% elimination of *methicillin-resistant S. aureus* (*MRSA*), and reduced retained crystal-violet-stained MRSA biofilm biomass by 78.57 ± 2.38% under the tested treatment and washing conditions. Multi-omics analysis identified treatment associated changes in central metabolism and nutrient transport. In an *MRSA*-infected mouse wound model, visible-light-activated TCN/GDY reduced the bacterial burden by 97.98 ± 1.04% and decreased the residual wound area to 2.93 ± 1.46% by day 11. This study establishes a graphdiyne-enabled TiO_2_/g-C_3_N_4_-based bio-heterojunction as a visible-light antibacterial photocatalytic platform for infected wound healing.

## 1 Introduction

The skin functions as the body's primary protective barrier, safeguarding internal organs from the external environment. Infected skin wounds remain difficult to manage because persistent bacterial colonization and biofilm formation prolong inflammation, delay re-epithelialization, and reduce the effectiveness of conventional therapies [1]. The rapid rise of antimicrobial resistance, especially *methicillin-resistant Staphylococcus aureus* (*MRSA*), further weakens antibiotic-centered treatment and increases the demand for non-antibiotic anti-infective strategies [2], [3].

Exogenous antibacterial strategies—including photothermal therapy (PTT), photodynamic therapy (PDT), and sonodynamic therapy (SDT), have garnered significant interest as promising alternatives [4], [5], [6]. Photocatalytic antibacterial systems are attractive in this context because they generate reactive oxygen species (ROS) on demand at the infected site, thereby providing localized bactericidal stress without systemic drug exposure [7], [8], [9]. The regulation of ROS concentration also plays a dual role in wound healing. During the proliferative phase, moderate ROS levels promote the recruitment of neutrophils and macrophages for tissue debridement and stimulate collagen and extracellular matrix (ECM) synthesis. Conversely, excessive ROS accumulation induces oxidative stress, which impairs the healing process [10], [11], [12]. Balancing antibacterial oxidative stress with host-tissue tolerance is therefore important when using ROS based treatment for infected wounds.

For wound related applications, visible light responsive photocatalysts offer a means of local antibacterial treatment [13], [14], [15]. The materials challenge is to achieve sufficient visible light harvesting, carrier separation, and ROS generation within a biologically acceptable treatment window. Excitation wavelength alone does not exclude heating; thermal effects depend on the irradiation conditions and material response [16], [17].

TiO_2_ is a classical photocatalyst with high physicochemical stability and photocatalytic activity, and unique n-type semiconductor characteristics. But its practical use under visible light is limited by a wide bandgap and rapid electron-hole recombination. Coupling TiO_2_ with graphitic carbon nitride is a useful route to broaden light absorption and improve band alignment [18], whereas graphdiyne (GDY) offers a conjugated sp/sp^2^ carbon framework with high conductivity and efficient electron delocalization [19]. Beyond these electronic advantages, Ti-based materials and GDY-containing systems have already shown promise in biomedical environments, which makes a GDY-enabled TiO_2_/CN interface attractive for antibacterial wound therapy [20].

In this work, “bio-heterojunction” is used as an application-oriented descriptor for the TiO_2_/g-C_3_N_4_/GDY heterojunction designed for photocatalytic treatment at a biological interface (Scheme 1). Here, “bio” refers to its intended use at an exposed infected wound surface and to the evaluation of its interactions with bacteria, mammalian cells, blood, and wound tissues. It does not refer to an interface containing living biological components or define a separate semiconductor junction class. TiO_2_/g-C_3_N_4_ provides the heterojunction scaffold, while GDY contributes to interfacial electronic coupling and carrier delocalization. The material was evaluated through structural and photoelectrochemical characterization, valence-band analysis, *in situ* XPS, EPR, DFT calculations, antibacterial and antibiofilm assays, multi-omics analysis, and an *MRSA*-infected wound model.

**Scheme 1:**
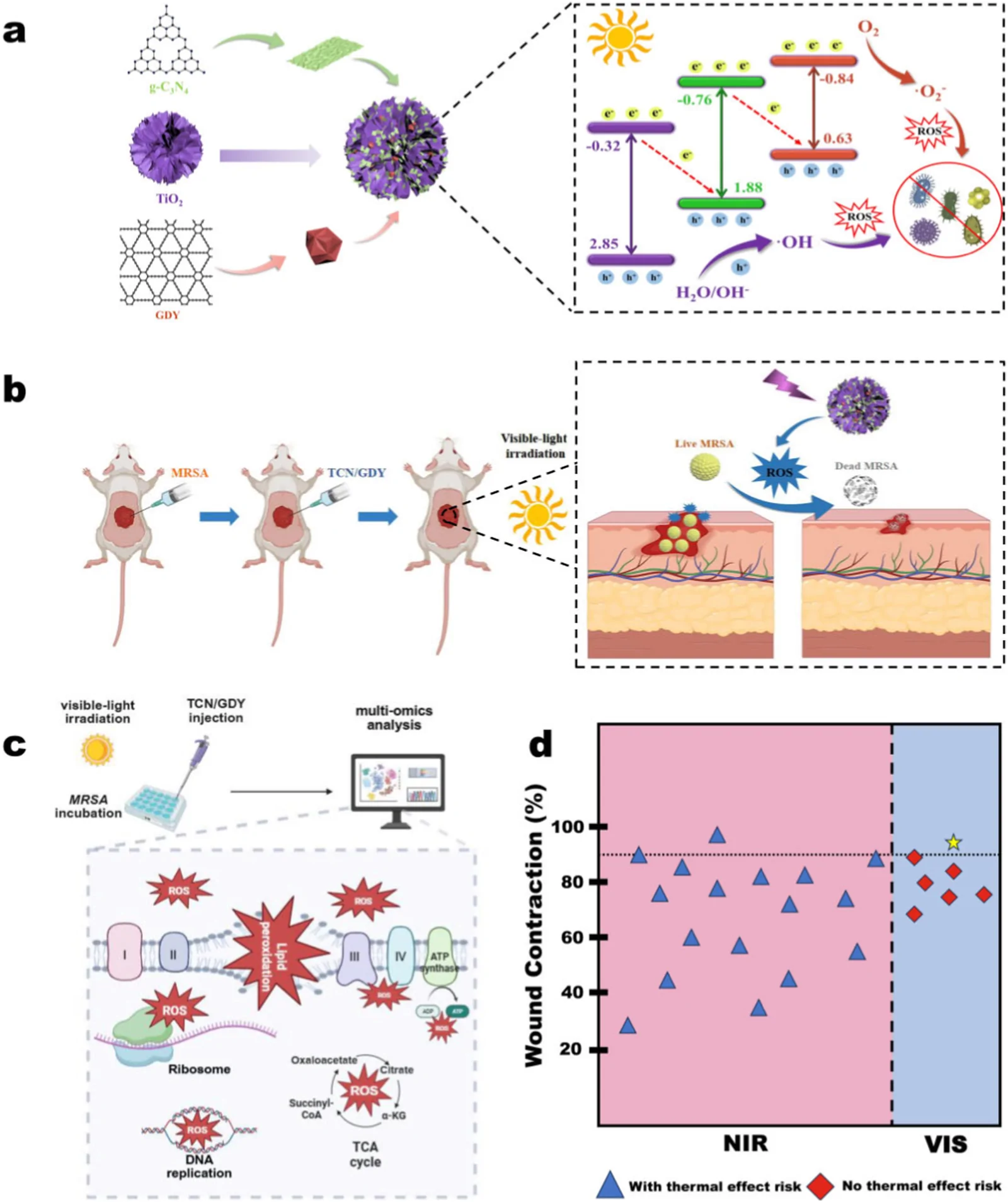
Design, working principle, and proposed therapeutic mechanism of the TCN/GDY bio-heterojunction under visible light. (a) Proposed dual S-scheme like interpretation of interfacial carrier behavior and ROS generation in TCN/GDY. (b) Proposed treatment process of *MRSA*-infected wounds using visible light activated TCN/GDY. (c) Multi-omics workflow and representative treatment associated molecular responses. (d) Descriptive comparison of reported wound healing outcomes across irradiation settings.

## 2 Results

### 2.1 Construction and morphology of the graphdiyne enabled TiO_2_/g-C_3_N_4_ based bio-heterojunction

Fig. 1a outlines the construction of the graphdiyne enabled TiO_2_/g-C_3_N_4_-based bio-heterojunction. CN was first obtained by thermal polymerization of urea, and TiO2 was prepared by a sonication-assisted hydrothermal process using tetrabutyl titanate in acetic acid. The binary TiO_2_/g-C_3_N_4_ scaffold (TCN) was then formed by ethanol-assisted mixing followed by annealing. Finally, GDY was introduced onto this scaffold through a CuCl-mediated deprotection-free growth process using HEB-TMS as the precursor. In this synthetic sequence, TiO_2_/g-C_3_N_4_ provides the base heterojunction, and GDY introduces a conjugated carbon component whose interfacial electronic contribution is examined below. CuCl acts as the coupling catalyst during GDY growth, after which the product was repeatedly washed with DMF, ethanol, and water before characterization and biological evaluation.

**Fig. 1:**
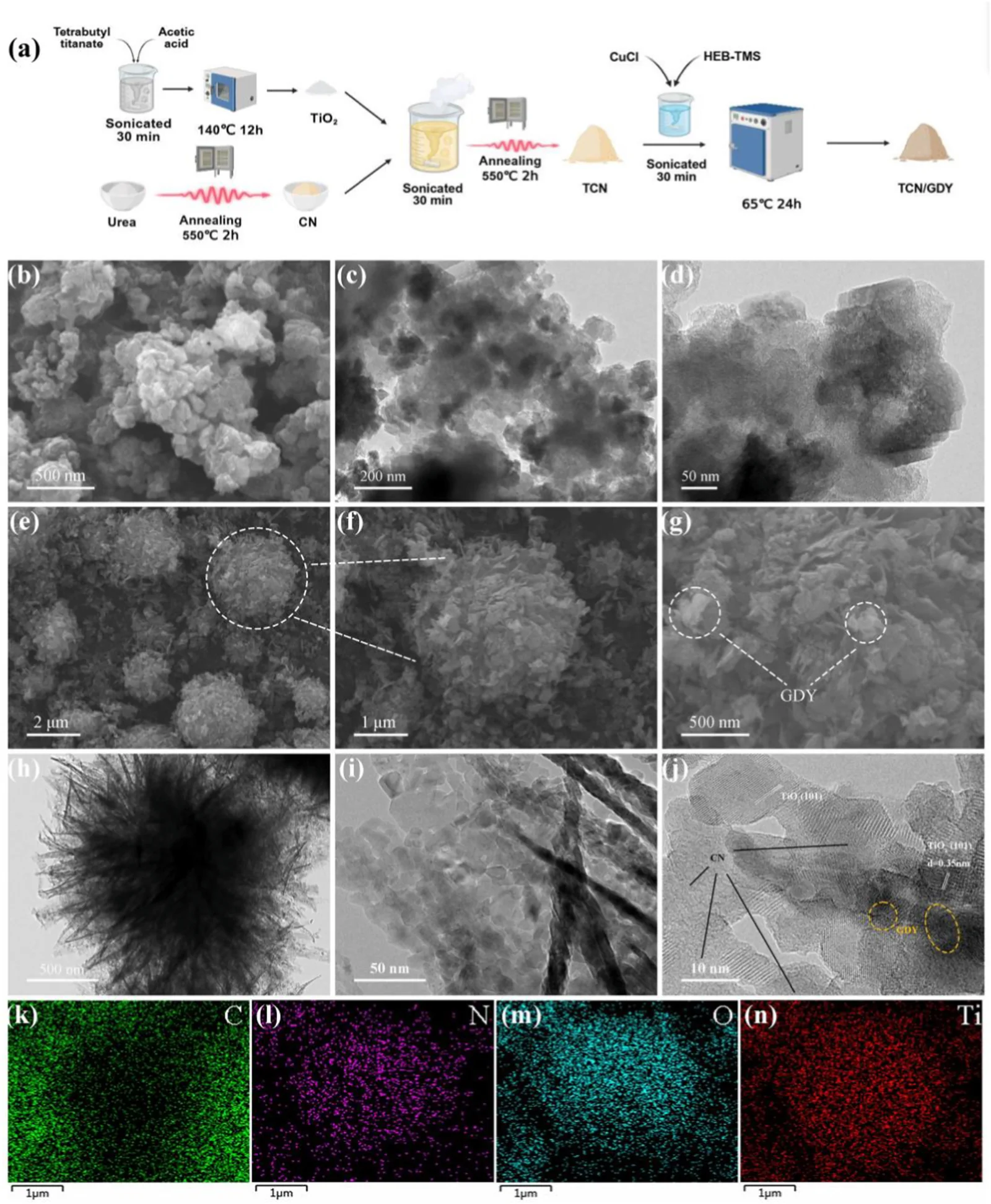
Morphology and elemental distribution of GDY and TCN/GDY. (a) Synthesis process of TCN/GDY. (b) SEM image of GDY. (c, d) TEM images of GDY. (e-g) SEM images of TCN/GDY. (h-j) TEM/HRTEM images of TCN/GDY. (k-n) EDS elemental maps of C, N, O, and Ti in TCN/GDY.

Scanning electron microscopy showed stacked features of GDY (Fig. 1b), whereas TCN/GDY retained the urchin-like morphology of the TiO_2_-containing scaffold (Fig. 1e–g). Transmission electron microscopy revealed layered GDY structures (Fig. 1c and d) and the composite morphology of TCN/GDY (Fig. 1h and i). A lattice spacing of approximately 0.35 nm was assigned to the anatase TiO_2_ (101) plane, alongside carbon-rich interfacial regions in the high resolution image (Fig. 1j). EDS maps showed spatially distributed C, N, O, and Ti signals within the examined composite region (Fig. 1k–n), supporting the coexistence of these elements in TCN/GDY. However, the absolute loading amount could not be reliably quantified because GDY forms *in situ* and is tightly hybridized with TCN at a low content. Future studies ought to address this concern.

### 2.2 Structural and interfacial chemical characterization

X-ray diffraction confirmed that TiO_2_ in T, TCN, and TCN/GDY remained in the anatase phase (Fig. 2a). The characteristic reflections at 25.3°, 37.9°, 48.0°, 54.0°, 55.2°, 62.7°, 68.8°, 70.3°, and 75.0° were assigned to the (101), (004), (200), (105), (211), (204), (116), (220), and (215) planes, respectively. GDY displayed a broad weak feature near 23°, consistent with an amorphous carbon rich framework. The preserved anatase pattern indicates that introduction of CN and GDY does not disrupt the TiO_2_ crystal structure.

**Fig. 2:**
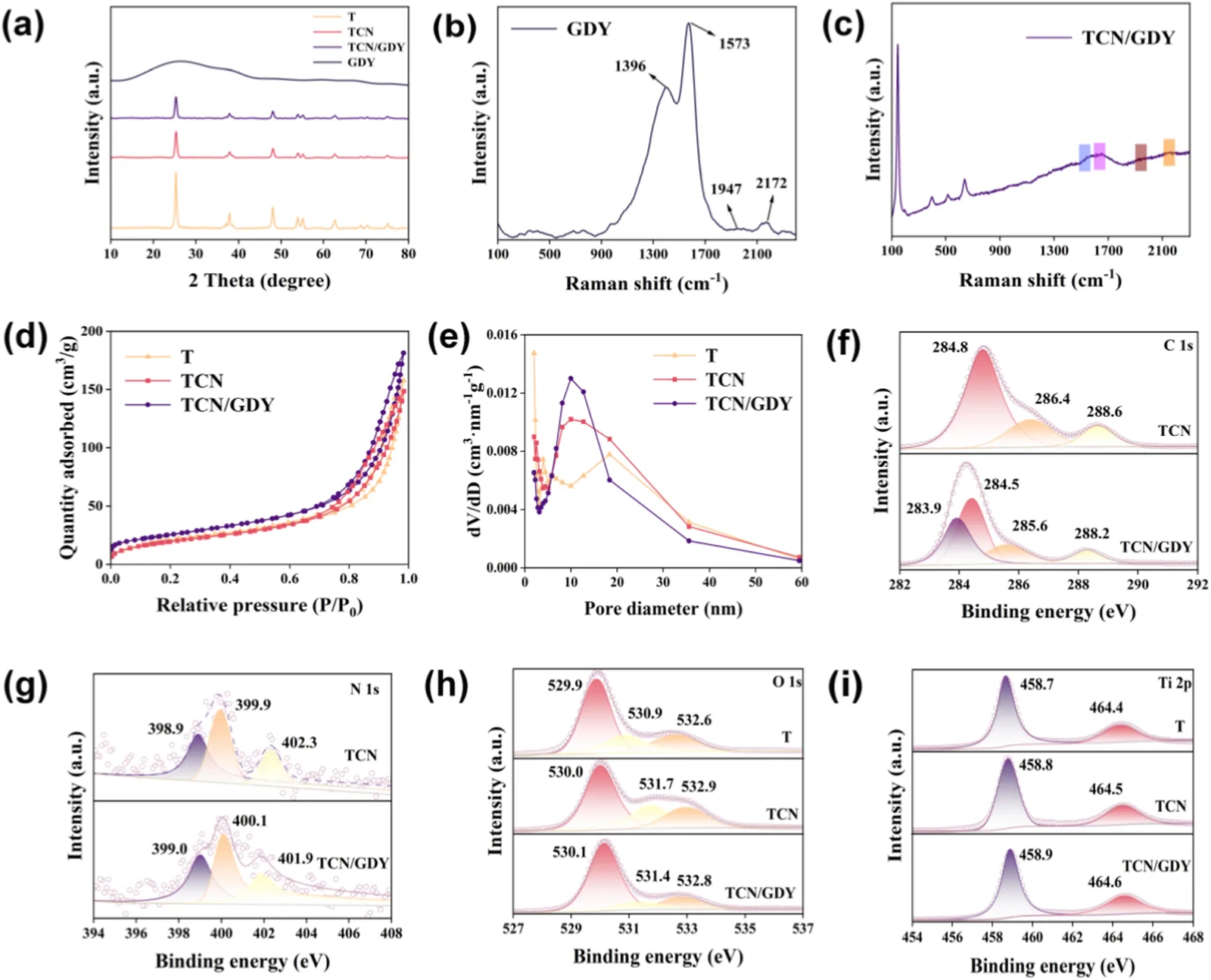
Structural and interfacial chemical characterization of GDY, T, TCN, and TCN/GDY. (a) XRD patterns. (b, c) Raman spectra of GDY and TCN/GDY. (d) N_2_ adsorption desorption isotherms. (e) Pore-size distribution curves. High-resolution XPS spectra of (f) C 1s and (g) N 1s for TCN and TCN/GDY, and (h) O 1s and (i) Ti 2p for T, TCN, and TCN/GDY.

Raman spectroscopy provided direct evidence for GDY incorporation (Fig. 2b, c and Fig. S1). Pure GDY exhibited the defect-related D band at about 1396 cm^−1^, the graphitic G band at about 1573 cm^−1^, and the characteristic conjugated diacetylene vibrations (—C ≡ C—) near 1947 and 2172 cm^−1^ [21]. T and TCN displayed the expected anatase TiO_2_ modes at approximately 144, 394, 514, and 636 cm^−1^ [22]. In TCN/GDY, the anatase bands coexisted with the GDY signatures, which supports successful integration of GDY onto the TiO_2_/g-C_3_N_4_ scaffold.

FTIR spectroscopy was used to examine the framework related vibrations and functional groups of T, TCN, and TCN/GDY (Fig. S2) [23], [24]. The broad low wavenumber absorption within 400–700 cm^−1^ is consistent with contributions from Ti–O and Ti–O–Ti lattice vibrations. TCN/GDY exhibits a band near 809 cm^−1^ and multiple bands within 1200–1650 cm^−1^, consistent with ring deformation and overlapping C–N/C=N stretching contributions associated with the carbon nitride framework. The broad envelope within 3000–3500 cm^−1^ may contain overlapping O–H and N–H stretching contributions, with adsorbed water contributing to the O–H absorption. Features within 2800–3000 cm^−1^ are consistent with aliphatic C–H stretching. Together, these observations provide complementary evidence for carbon-nitride related structural motifs in TCN/GDY and information on the functional groups present in the samples.

N_2_ adsorption-desorption measurements showed type IV isotherms with H3 hysteresis loops for T, TCN, and TCN/GDY, consistent with mesoporosity (Fig. 2d and e). The dominant pore diameters were 18.68 nm for T, 10.31 nm for TCN, and 9.98 nm for TCN/GDY. The specific surface area increased from 70.54 m^2^ g^−1^ for T to 105.09 m^2^ g^−1^ for TCN and 121.68 m^2^ g^−1^ for TCN/GDY, indicating that CN integration and subsequent GDY growth increase accessible interfacial area and provide more active sites for photocatalytic reactions.

X-ray photoelectron spectroscopy (XPS) further clarified the chemical states and interfacial electronic interactions. Following the introduction of GDY, a significant enhancement in the C 1s signal intensity is observed for TCN/GDY (Fig. S3), consistent with the successful incorporation of the carbonaceous component into the heterostructure. The C 1s spectrum for TCN can be deconvoluted into three contributions at 284.8, 286.4, and 288.6 eV, assigned to sp^2^-hybridized carbon (C=C/C–C), incompletely condensed C–NHx species at aromatic edges, and sp^2^ carbon in the aromatic C–N framework, respectively. In contrast, the C 1s spectrum for TCN/GDY resolved four peaks: sp-hybridized C ≡ C (283.9 eV), sp^2^-hybridized C=C (284.5 eV), C–O (285.6 eV), and aromatic C–N (288.2 eV) [25]. The appearance of the characteristic C ≡ C peak and the shift in sp^2^-carbon signal provide direct evidence for the successful integration of GDY into the composite structure. In N 1s spectra, the spectrum can be deconvoluted into three peaks located at 398.9, 399.9, and 402.3 eV for TCN, corresponding to sp^2^-hybridized N in the aromatic C–N framework, sp^3^-hybridized N at the tri-s-triazine ring center, and incompletely condensed N at the edges of aromatic structures, respectively. A distinct shift in binding energy was observed for all N-related peaks for TCN/GDY, indicating that the introduction of GDY alters the electronic environment of nitrogen. These spectral changes are consistent with altered interfacial electronic environments after GDY incorporation [26]. The high-resolution O 1s XPS spectra are shown in Fig. 2h. For T, the spectrum can be fitted with three peaks: peaks at binding energies of 529.9 eV, 530.9 eV, and 532.6 eV correspond to Ti–O chemical bonds, adsorbed oxygen, and adsorbed water, respectively. TCN and TCN/GDY also show three similar peaks, with the peaks for TCN/GDY at 531.4 eV and 532.8 eV attributed to C–O and C=O chemical bonds [27]. The high-resolution Ti 2p spectra of T, TCN, and TCN/GDY contain the spin-orbit components associated with Ti (IV) (Fig. 2i) [28]. Their spectral differences are consistent with changes in the local Ti electronic environment after composite formation. Together, the component spectra support interfacial electronic interaction.

### 2.3 Visible light photoelectrochemical behavior and band structure analysis

TCN/GDY showed the largest transient photocurrent response among the tested samples, reaching approximately 1.89 μA cm^−2^ over repeated light on/off cycles (Fig. 3a). It also showed the smallest EIS Nyquist semicircle and the lowest PL intensity in series T, TCN, and TCN/GDY (Fig. 3b and c). Together, these observations are consistent with improved interfacial charge transport and a changed balance of carrier recombination under the measured conditions. They support the enhanced photoresponse.

**Fig. 3:**
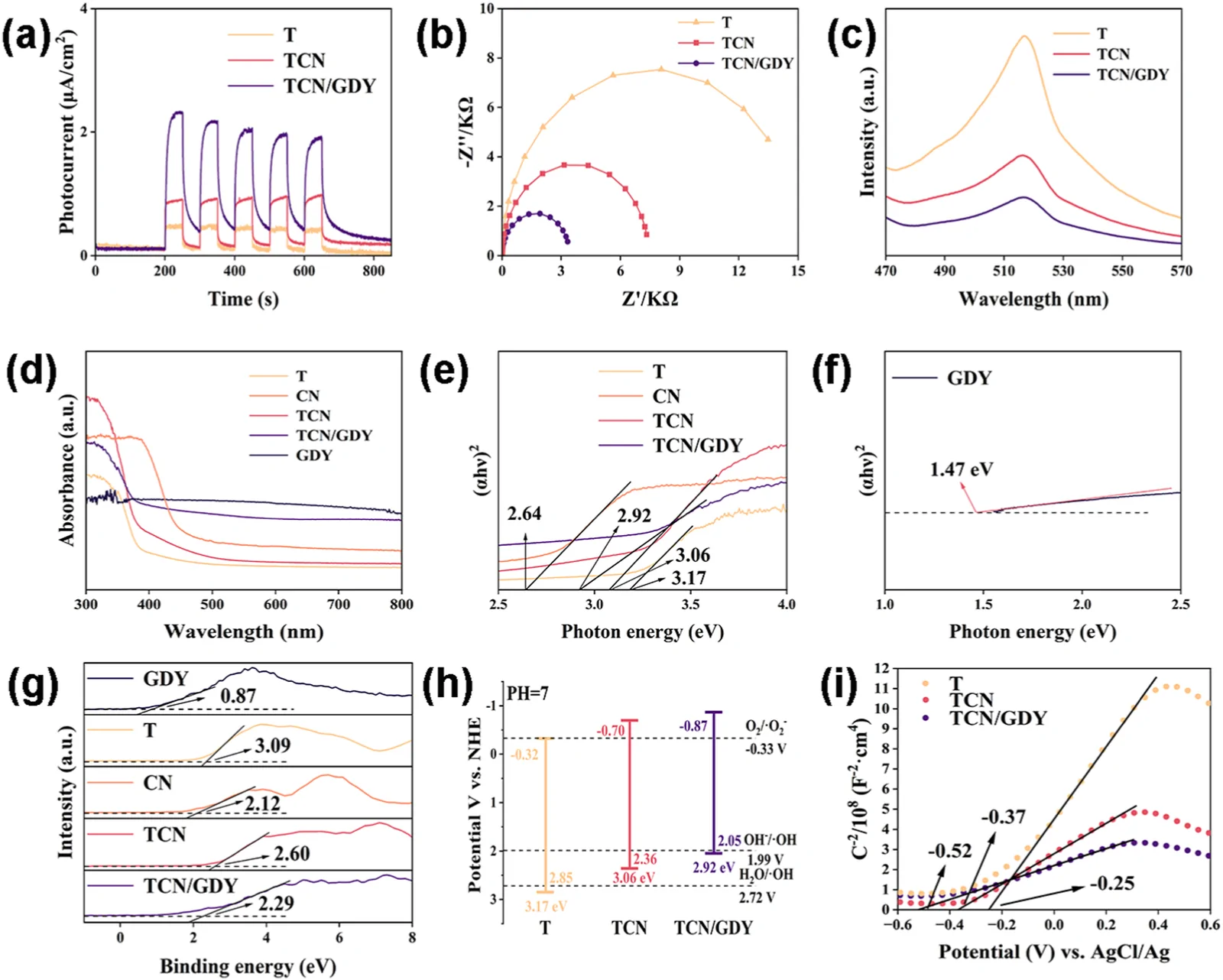
Photoelectrochemical properties and band-structure analysis of T, TCN, and TCN/GDY. (a) Transient photocurrent responses. (b) Electrochemical impedance spectroscopy (EIS) Nyquist plots. (c) Photoluminescence (PL) spectra. (d) UV-vis diffuse reflectance spectra. (e, f) Tauc plots derived from the Kubelka-Munk transformation. (g) Valence-band XPS spectra. (h) Estimated band positions of T, TCN, and TCN/GDY. (i) Mott-Schottky plots.

UV-Vis diffuse reflectance spectra showed that pristine TiO_2_ absorbed mainly in the ultraviolet region, whereas TCN and especially TCN/GDY displayed broadened visible light absorption and red shifted absorption edges (Fig. 3d). Tauc analysis yielded optical bandgaps of 3.17 eV for T, 3.06 eV for TCN, and 2.92 eV for TCN/GDY, while the bandgaps of CN and GDY were 2.64 and 1.47 eV, respectively (Fig. 3e and f). The bandgap narrowing and broadened absorption window are favorable for visible light antibacterial photocatalysis.

Valence band XPS was used to estimate band positions (Fig. 3g). The measured valence band maxima were 0.87 eV for GDY, 2.12 eV for CN, 3.09 eV for T, 2.60 eV for TCN and 2.29 eV for TCN/GDY. After conversion to the normal hydrogen electrode scale, the corresponding valence band potentials were 0.63, 1.88, 2.85, 2.36 and 2.05 eV, respectively. Combining these values with the optical bandgaps provided the band alignment shown in Fig. 3h. Mott-Schottky plots further supported the n type character of the semiconductor components and were consistent with a band architecture capable of driving oxygen reduction and ROS-related oxidation processes under visible light (Fig. 3i).

### 2.4 DFT analysis of the graphdiyne enabled heterointerface

Ground-state DFT calculations were used to examine a selected, ordered TiO_2_/CN/GDY local interface (Fig. 4a and b). Its electronic structure depends on the chosen stacking and contact geometry. PBE/SZV-MOLOPT-GTH calculations are therefore used to interpret qualitative orbital contributions and interfacial coupling.

**Fig. 4:**
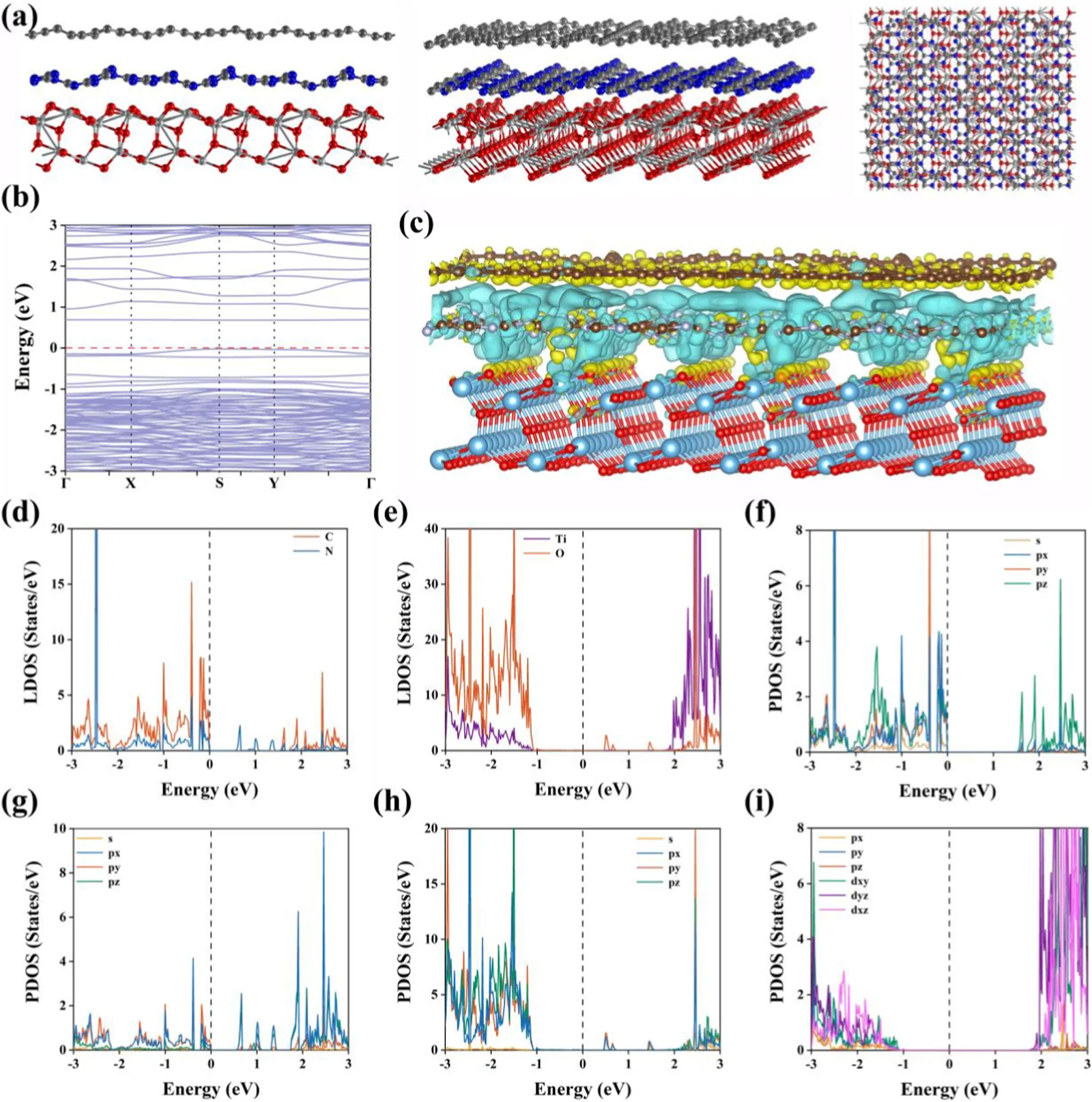
Ground-state DFT analysis of an idealized local TiO_2_/CN/GDY interface. (a) Ordered periodic structural model. (b) Calculated electronic band structure. (c) Charge-density-difference map. (d, e) LDOS of CN and TiO_2_, respectively. (f-i) Orbital-resolved PDOS: C, N, O, Ti.

The charge-density-difference map shows local electron accumulation and depletion upon interface formation (Fig. 4c). Quantitative comparison with the frozen isolated components gives real-space electron-number changes of +1.305, −3.122, and +1.816 electrons per simulated supercell for TiO_2_, CN, and GDY, respectively, where positive values denote electron gain (Fig. S4 and Table S1). The Mulliken and Hirshfeld partitions give the same signs, and the real-space signs remain unchanged when the integration boundaries are shifted by ±0.3 Å. These results describe CN electron depletion and electron gain in TiO_2_ and GDY within the selected ground-state model.

The PDOS profiles show C 2p, N 2p and O 2p, Ti 3d contributions to the calculated electronic structure (Fig. 4f-i). The CN, TiO_2_ and GDY LDOS are shown in Fig. 4d, e and Fig. S5, respectively. These orbital projections support interfacial electronic coupling within the model. The side-resolved isolated-component work functions, defined as Φ = E_vac_ − E_F_, place CN below TiO_2_ and GDY on both vacuum sides (Fig. S6). The relative ordering of TiO_2_ and GDY depends on the vacuum side because the slabs are asymmetric. The contact-induced electrostatic response is nonuniform across the two interfaces. These model-dependent quantities support contact polarization without implying a single serial electron-transfer sequence through all three components.

### 2.5 *In-situ* charge transfer behavior and ROS generation

Dark and illuminated XPS spectra were collected from the same specimen at the same sampling position (Fig. 5a–d). For all 12 labeled components, ΔBE = BE_light_ − BE_dark_ is −0.53 to −0.26 eV for C 1s, +0.12 to +0.24 eV for N 1s, and +0.15 to +0.26 eV for Ti 2p; the two O 1s shifts are +0.15 and −0.52 eV (Fig. S7 and Table S2). Carbon is present in both CN and GDY, so the C 1s shifts are not assigned exclusively to GDY. The main Ti, N, and O peak separations increase by +0.58, +0.47, and +0.47 eV, respectively (Table S3). This supports a nonuniform spectral response.

**Fig. 5:**
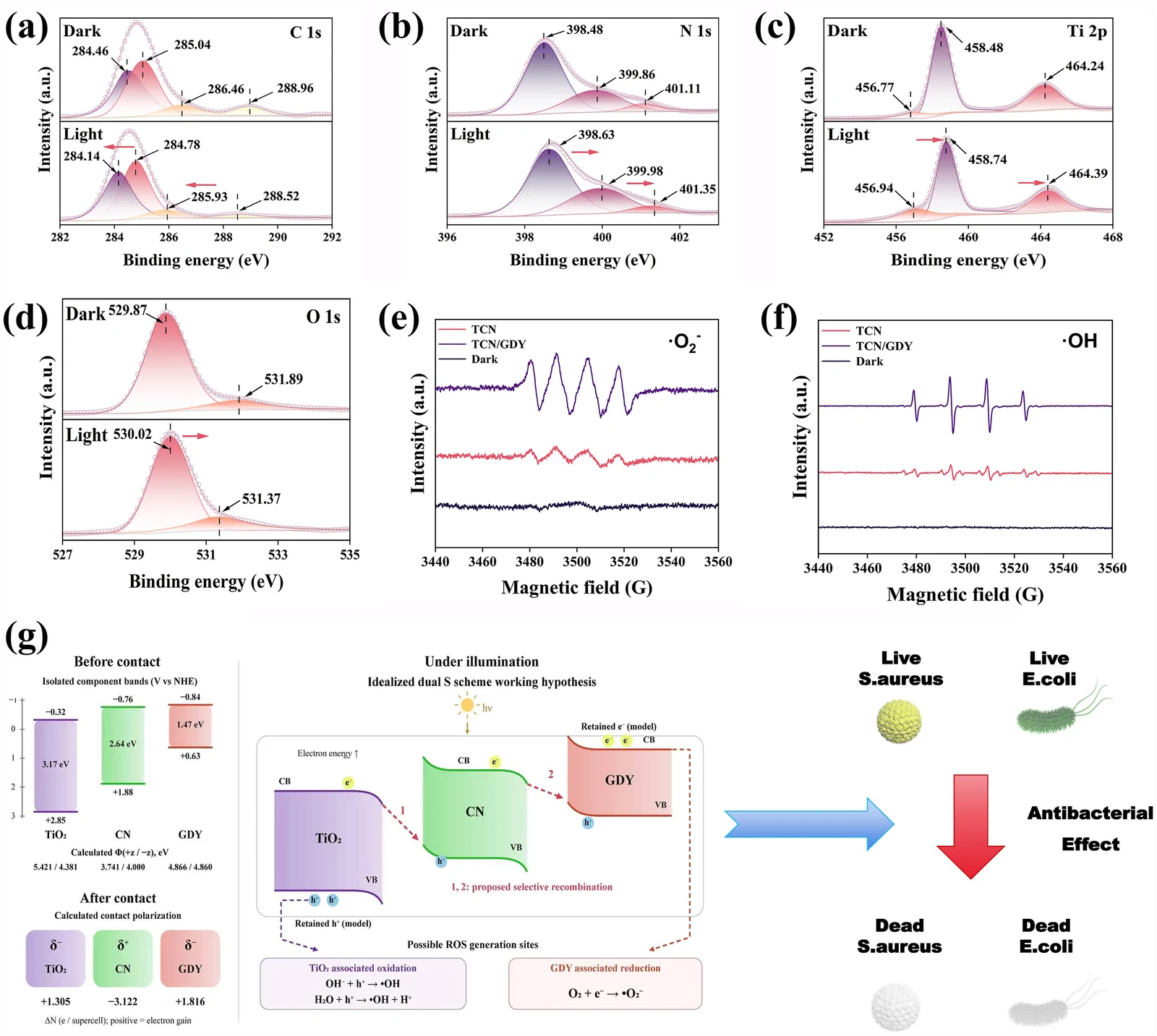
Illumination-dependent XPS spectra and radical-adduct EPR signals of TCN/GDY. (a-d) High-resolution C 1s, N 1s, Ti 2p, and O 1s spectra recorded in the dark and under visible-light irradiation. (e, f) EPR spectra of DMPO-superoxide and DMPO-hydroxyl radical adducts for TCN and TCN/GDY. (g) Proposed dual S-scheme like interpretation.

The illumination-dependent XPS changes indicate an altered electronic environment; radical generation is assessed separately by EPR. Formaldehyde degradation was used as a probe of photocatalytic activity under the reported visible-light conditions (nominal source power, 3 W). The concentration profiles in Fig. S8 show a faster decrease for TCN/GDY than for T and TCN, with the displayed TCN/GDY concentration approaching zero. The plotted record extends over 12 h. These results support formaldehyde removal under the assay conditions.

The apparent pseudo-first-order rate constants are 0.119 min^−1^ for TCN/GDY and 0.025 min^−1^ for T, corresponding to a 4.76-fold difference (Figs. S9 and S10). These rate constants characterize formaldehyde disappearance in this assay, which is equal to the ROS generation rates. The photoelectrochemical and EPR comparisons support an enhanced photoresponse and radical formation in TCN/GDY under the measured conditions. Fig. S11 shows an extended low formaldehyde concentration interval followed by repeated concentration increases and decreases associated with renewed formaldehyde challenges. The repeated decreases support continued formaldehyde removal after readdition under the recorded conditions, confirming establish uninterrupted ROS generation throughout the preceding 96 h.

The stronger DMPO-superoxide and DMPO-hydroxyl radical-adduct EPR signals of TCN/GDY (Fig. 5e and f), together with its enhanced photocurrent, smaller EIS semicircle, and lower steady-state PL intensity, support improved photocarrier utilization and radical generation. To rationalize these observations, a dual S-scheme charge transfer mechanism is proposed in Fig. 5g. TiO_2_ conduction band electrons are envisaged to recombine with CN valence band holes through channel 1, while CN conduction band electrons recombine with GDY valence band holes through channel 2. This interpretation retains reducing electrons on GDY and oxidizing holes on TiO_2_, providing possible routes for O_2_ reduction to •O_2_^−^ and OH^−^ oxidation to •OH.

### 2.6 *In vitro* antibacterial and antibiofilm performance

Given the high ROS-generation efficiency of the TCN/GDY bio-heterojunction, we further evaluated its broad-spectrum antibacterial performance. Photocatalytic antibacterial assays were conducted against *E. coli*, *S. aureus*, and *MRSA* under both 420 nm LED and xenon-lamp irradiation for 30 min (Fig. 6a and b and Figs. S12–S15). As shown in Fig. 6a, in the dark, T, TCN, and TCN/GDY showed negligible antibacterial activity compared to the control. Furthermore, the residual Cu in TCN/GDY was evaluated using ICP-OES (Fig. S16). The results indicated that residual Cu of 0.056 mg∙L^−1^ or released Cu of 0.045, 0.051 mg∙L^−1^ without or with xenon light irradiation in 24h did not contribute significantly to the antibacterial activity, confirming that their antibacterial effect is light-dependent.

**Fig. 6:**
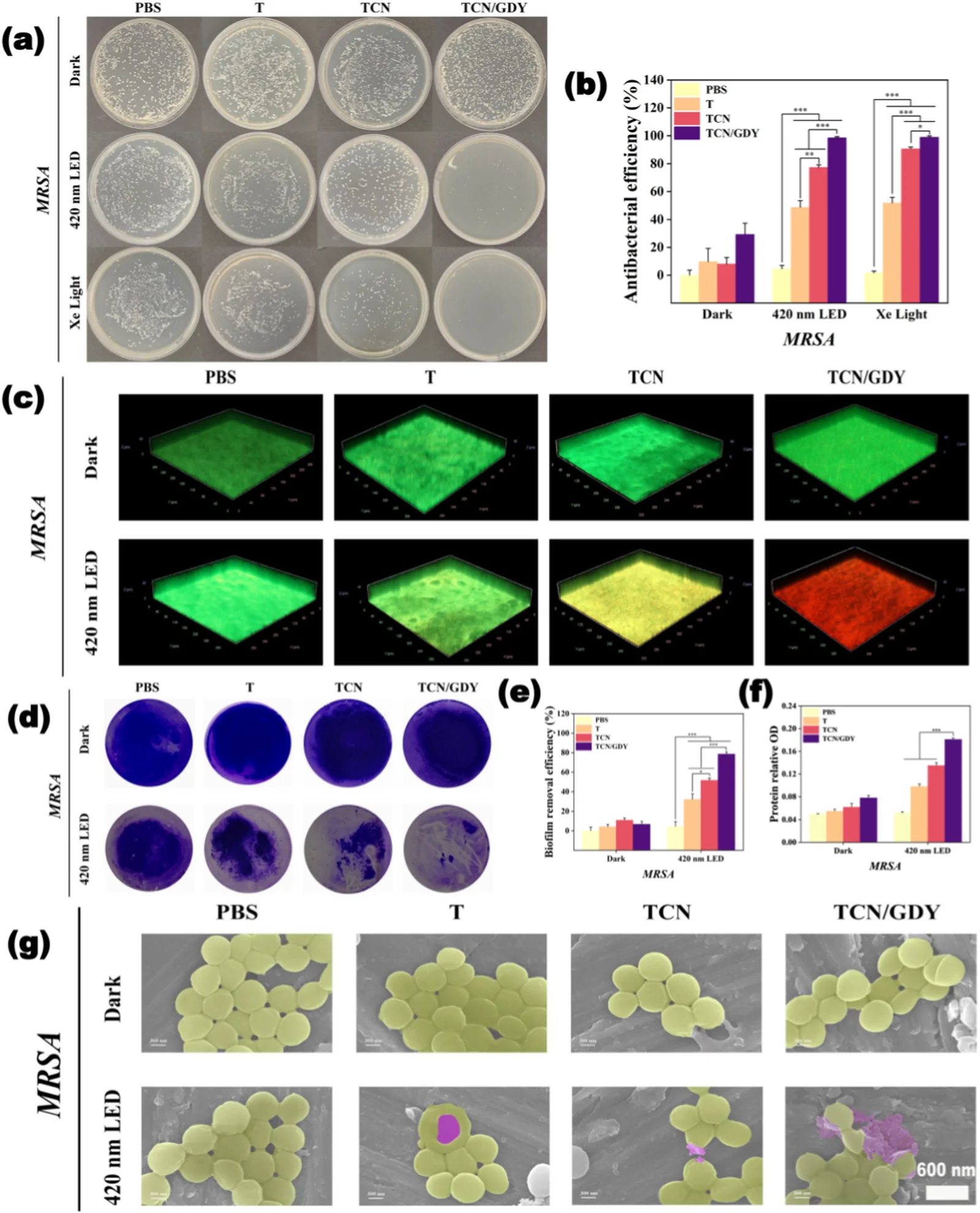
Visible light driven antibacterial and antibiofilm performance of TCN/GDY. (a) Representative MRSA colony images after treatment with PBS, T, TCN, or TCN/GDY in the dark or under 420 nm LED or Xe-lamp irradiation. (b) Corresponding antibacterial efficiencies determined from recoverable CFU counts. (c) SYTO9/PI fluorescence images of *MRSA* biofilms. (d) Representative crystal violet-stained biofilms. (e) Reduction in crystal violet-stained retained biofilm biomass after treatment and washing. (f) Extracellular protein leakage from MRSA. (g) Representative FESEM images of treated MRSA. Quantitative data in panels b, e, and f are presented as mean ± SD (n = 3; *p < 0.05, **p < 0.01, ***p < 0.001).

Upon irradiation, a progressive reduction in bacterial viability was observed across all samples, indicating varying levels of photocatalytic antibacterial activity. Under LED light, the antibacterial rates of T, TCN, and TCN/GDY against *E. coli* reached 85.36 ± 4.29%, 96.62 ± 2.47%, and >99%, respectively. Under xenon-lamp irradiation, the corresponding rates were 59.93 ± 3.31%, 85.60 ± 1.22%, and 99.84 ± 0.11%. In all cases, the TCN/GDY bio-heterojunction achieved the highest antibacterial efficiency, which is attributed to its superior ROS-generating capacity under visible light, leading to effective bacterial inactivation (Figs. S14 and S15). As shown in Fig. 6a, b and Figs. S12 and S13, under LED irradiation, the antibacterial rates of T, TCN, and TCN/GDY against *S. aureus* were 54.53 ± 4.73%, 66.91 ± 2.25%, and 99.40 ± 0.34%, respectively, while against *MRSA* the rates reached 48.61 ± 4.87%, 77.28 ± 1.88%, and 98.65 ± 0.74%. Under xenon lamp irradiation, the corresponding antibacterial rates against *S. aureus* were 58.05 ± 7.69%, 73.39 ± 2.64%, and 99.75 ± 0.03%, and against *MRSA* were 51.75 ± 4.05%, 90.71 ± 1.29%, and 99.01 ± 0.89%. In summary, the ternary TCN/GDY bio-heterojunction, formed by coupling CN and GDY, exhibited potent and consistent antibacterial activity against Gram-negative (*E. coli*), Gram-positive (*S. aureus*), and drug-resistant (*MRSA*) bacteria under both LED and xenon-light sources, confirming its broad-spectrum photocatalytic performance and adaptability to different visible-light environments.

Fig. 6c shows live/dead staining images of *MRSA* biofilms observed by confocal microscopy, where live and dead bacteria were labeled green and red, respectively. Under dark conditions, all groups displayed predominantly green fluorescence, confirming negligible antibacterial activity in the absence of light. Following visible-light irradiation, the red fluorescence intensity progressively increased across the samples. The T group showed light-green fluorescence resulting from a mixture of weak red and abundant green signals. The TCN group exhibited yellow-tinged fluorescence, indicating a combination of residual green and dominant red fluorescence. In contrast, the TCN/GDY sample displayed extensive red fluorescence with only sparse green spots, demonstrating substantial bacterial killing and effective biofilm disruption under visible-light photocatalysis. The anti-biofilm efficacy against *MRSA* was quantified using crystal violet staining (Fig. 6d). In the dark, none of the samples significantly disrupted pre-formed biofilms. Upon visible-light irradiation, biofilm clearance increased progressively, with rates of 32.01 ± 5.74% for T, 51.69 ± 2.18% for TCN, and 78.57 ± 2.38% for TCN/GDY (Fig. 6e). Protein leakage assays confirmed significantly elevated extracellular protein levels in the TCN/GDY group following visible-light irradiation (Fig. 6f), directly linking its superior ROS-generating capacity to severe membrane damage in *MRSA*. Morphological changes in *MRSA* were visualized by FESEM (Fig. 6g). In the dark, *MRSA* in all groups remained intact and smooth. After visible-light irradiation, T-treated bacteria showed partial surface depression, TCN-treated cells exhibited localized rupture, while TCN/GDY caused extensive cell disintegration with obvious leakage of intracellular contents (highlighted in purple), confirming its potent bactericidal effect. Collectively, the colony assays, fluorescence images and morphology observations support antibacterial activity and membrane damage, while crystal violet staining reports reduced retained biofilm biomass, demonstrating that the TCN/GDY bio-heterojunction possesses outstanding visible light driven antibacterial and antibiofilm activity, effectively disrupting bacterial integrity through ROS-mediated membrane damage and biomolecule leakage. The colony-count assays assess loss of culturability after the specified treatment. Time-resolved bacterial growth, inhibitory concentrations, and regrowth after treatment were not evaluated. Thus, our future studies will include growth inhibition assays to further assess its antibacterial properties.

### 2.7 Multi-omics analysis reveals antibacterial mechanisms

To investigate the antibacterial mechanism at the systems level, transcriptomic and metabolomic analyses were performed on *MRSA* after visible-light treatment (Fig. 7, Fig. 8a). Transcriptomic profiling (RNA-seq) of treated *MRSA* revealed high data reproducibility (Fig. 7b–d and Figure S17, S18) and identified 465 differentially expressed genes (DEGs: 161 up-regulated, 304 down-regulated; Fig. 7c–e and Fig. S19). Principal component analysis (PCA) further confirmed minimal intra-group variation alongside distinct inter-group separation, highlighting the reproducibility of samples within the same treatment (Fig. S18). KEGG enrichment analysis highlighted severe disruption in central metabolic and regulatory pathways. Key pathways included the citrate (TCA) cycle and peroxisome function, indicating impaired energy production and oxidative stress management. Concurrently, the two-component system (TCS) and phosphotransferase system (PTS), critical for environmental sensing, nutrient uptake, and bacterial proliferation—were markedly suppressed (Fig. 7f). GO analysis further revealed significant alterations in cellular homeostasis and amino-acid-binding processes (Fig. 7g), reflecting a broad breakdown of intracellular steady-state and biosynthetic capacity. Furthermore, Clusters of Orthologous Groups (COG) functional annotation revealed that TCN/GDY bio-heterojunction significantly perturbed key biological processes in *MRSA*, most notably in Energy production and conversion, Amino acid transport and metabolism, and Inorganic ion transport and metabolism. Collectively, these transcriptomic perturbations illustrate that TCN/GDY-driven photocatalytic stress disrupts *MRSA* at multiple functional nodes: by blocking energy metabolism, compromising stress adaptation, and interfering with signal transduction and nutrient assimilation. This systemic dysregulation ultimately leads to growth inhibition and bacterial death, providing a molecular-level explanation for the observed antibacterial efficacy.

**Fig. 7:**
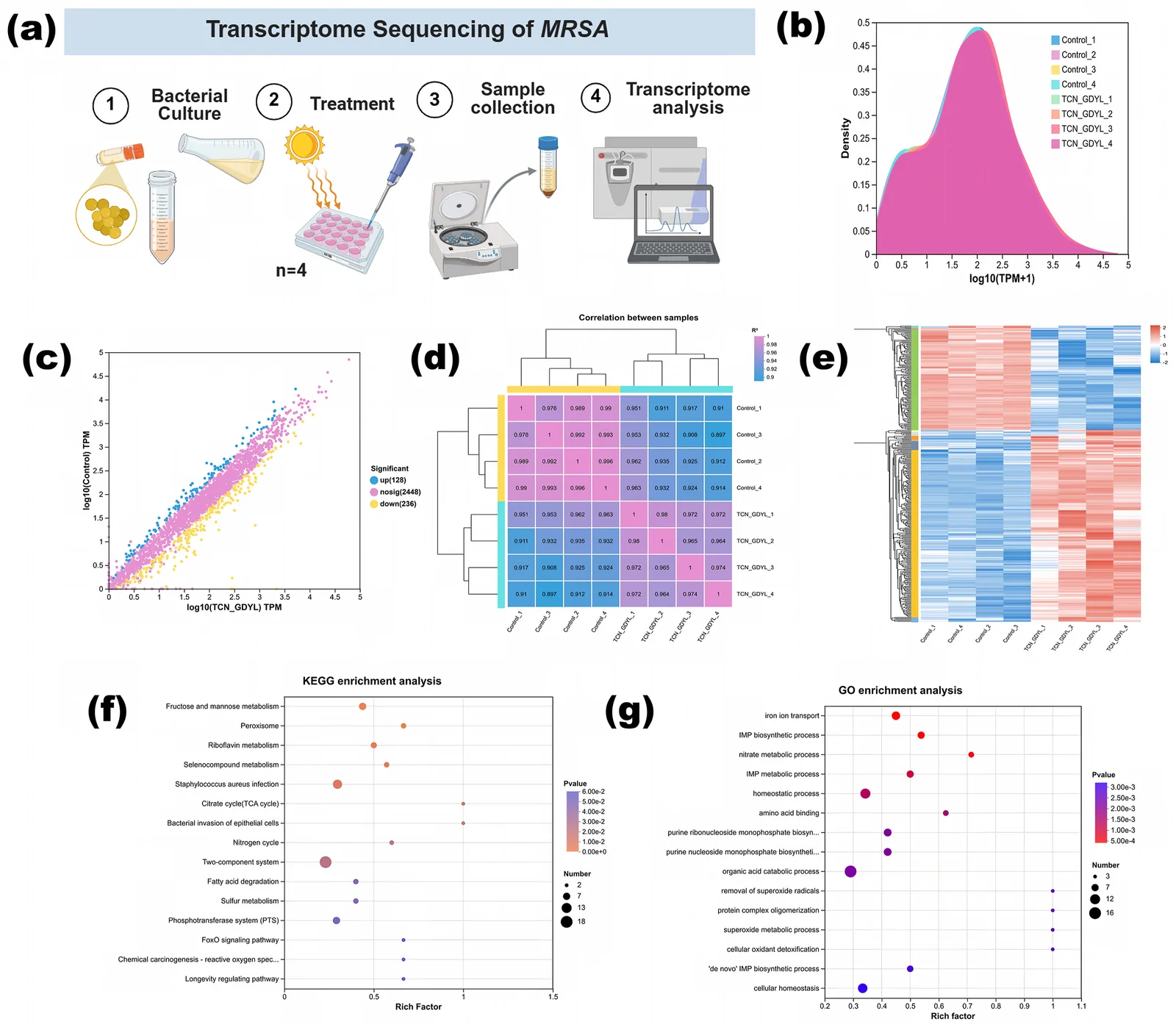
Transcriptomic analyses of *MRSA* after treatment with visible light activated TCN/GDY. (a) Schematic diagram of transcriptome sequencing of *MRSA*. (b) Transcripts per million (TPM) distribution across samples. (c) Differentially expressed genes (DEGs), divided into up- and down-regulated categories. (d) Correlation matrix of transcriptomic samples. (e) Heatmap of DEG expression. (f) Top 15 KEGG enrichment results for DEGs. (g) Top 15 GO enrichment results for DEGs. Data are presented as mean ± SD (n = 4, *p < 0.05, **p < 0.01, ***p < 0.001).

**Fig. 8:**
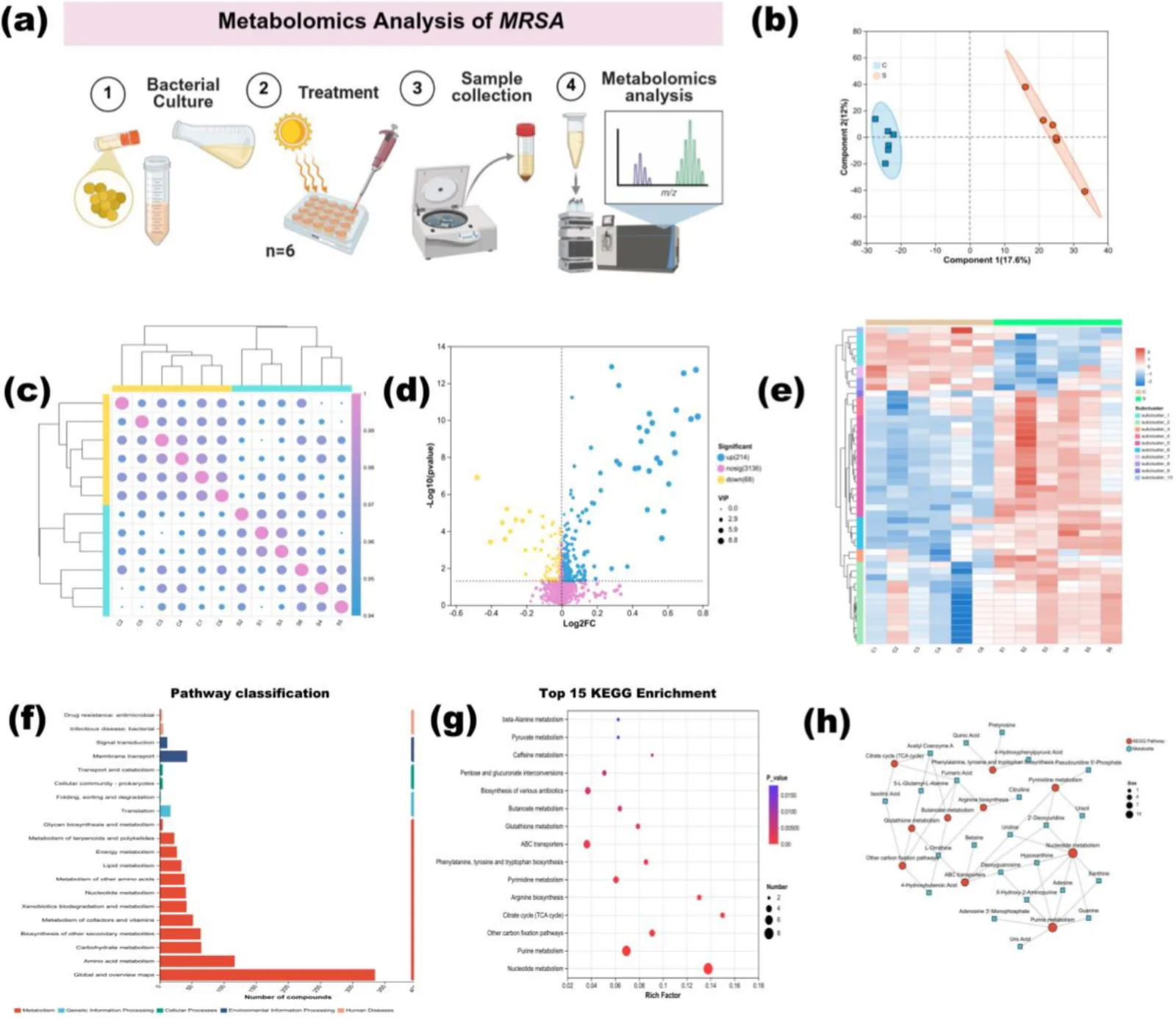
Metabolomic analyses of *MRSA* after treatment with visible light activated TCN/GDY. (a) Schematic diagram of metabolomics analysis of *MRSA.* (b) Partial least-squares discriminant analysis (PLS-DA) score plot. (c) Metabolomic sample correlation analysis. (d) Volcano plot of differentially accumulated metabolites (DAMs). (e) Heatmap of DAM abundance. (f) Pathway classification of DAMs. (g) Top 15 KEGG enrichment results for DAMs. (h) Network visualization of enriched metabolic pathways. Data are presented as mean ± SD (n = 6, *p < 0.05, **p < 0.01, ***p < 0.001).

Based on transcriptomic evidence that TCN/GDY significantly disrupts *MRSA* metabolism under visible-light irradiation, metabolomic profiling was further conducted to delineate the specific metabolic lesions (Fig. 8a). PLS-DA and correlation analysis confirmed data reproducibility (Fig. 8b and c). A total of 282 differentially accumulated metabolites (DAMs) were identified (214 upregulated, 68 downregulated; Fig. 8d). Cluster analysis revealed a marked downregulation of metabolites in the TCN/GDY-treated group (Fig. 8e), indicative of systemic metabolic suppression. Pathway analysis of DAMs highlighted profound disturbances in energy metabolism**,** lipid metabolism, and carbohydrate metabolism. KEGG enrichment further pinpointed critical disruption of the TCA cycle, pyruvate metabolism, and purine metabolism, all central to ATP production and bacterial energetics (Fig. 8f and g). Concurrently, pathways related to ABC transporters, key players in nutrient uptake, toxin extrusion, and bacterial signal transduction, were significantly altered, reflecting impaired transmembrane sensing and transport.

A network visualization of enriched pathways (Fig. 8h) integrated these changes, illustrating how TCN/GDY-induced photocatalytic stress simultaneously cripples energy generation and disrupts signal-transduction-linked transport systems. Furthermore, comparison of the transcriptomic and metabolomic pathway results identifies shared and complementary associations with bacterial metabolism. The TCA cycle is represented in both datasets (Fig. 7, Fig. 8g), linking the transcriptional and metabolic responses to central carbon metabolism. Transcriptomic GO terms related to IMP biosynthesis and purine nucleotide metabolism further complement the metabolomic associations with purine metabolism (Fig. 7, Fig. 8g). Associations with the two component and phosphotransferase systems at the transcriptomic level, together with ABC transporter related metabolomic findings, suggest additional changes in environmental sensing and nutrient handling. These observations provide a pathway level interpretation across the independently sampled datasets.

### 2.8 *In vivo* efficacy in an *MRSA* infected wound model

To investigate the *in vivo* antibacterial activity of the TCN/GDY bio-heterojunction *via* photocatalysis under visible light, we established an 8 mm dorsal skin defect model in BALB/c mice infected with methicillin-resistant *S. aureus* (*MRSA*) and evaluated the wound healing effects (Fig. 9a). As shown in Fig. 9b, c and e, wound healing progressed differently among the groups during the treatment period. From day 3 to day 11, the wound closure rates followed the order: PBS (−) < PBS (+) < TCN/GDY (−) < TCN/GDY (+). The TCN/GDY (+) group demonstrated the most pronounced healing effect, showing significantly enhanced tissue regeneration and superior wound closure compared to all other groups. By day 11, the wound area in the TCN/GDY (+) group was minimal, at only 2.93 ± 1.46%, while the wound areas in other groups remained above 10%. Fig. 9g recorded the body weight changes of the mice, which showed an overall increasing trend, indicating no adverse effects during treatment.

**Fig. 9:**
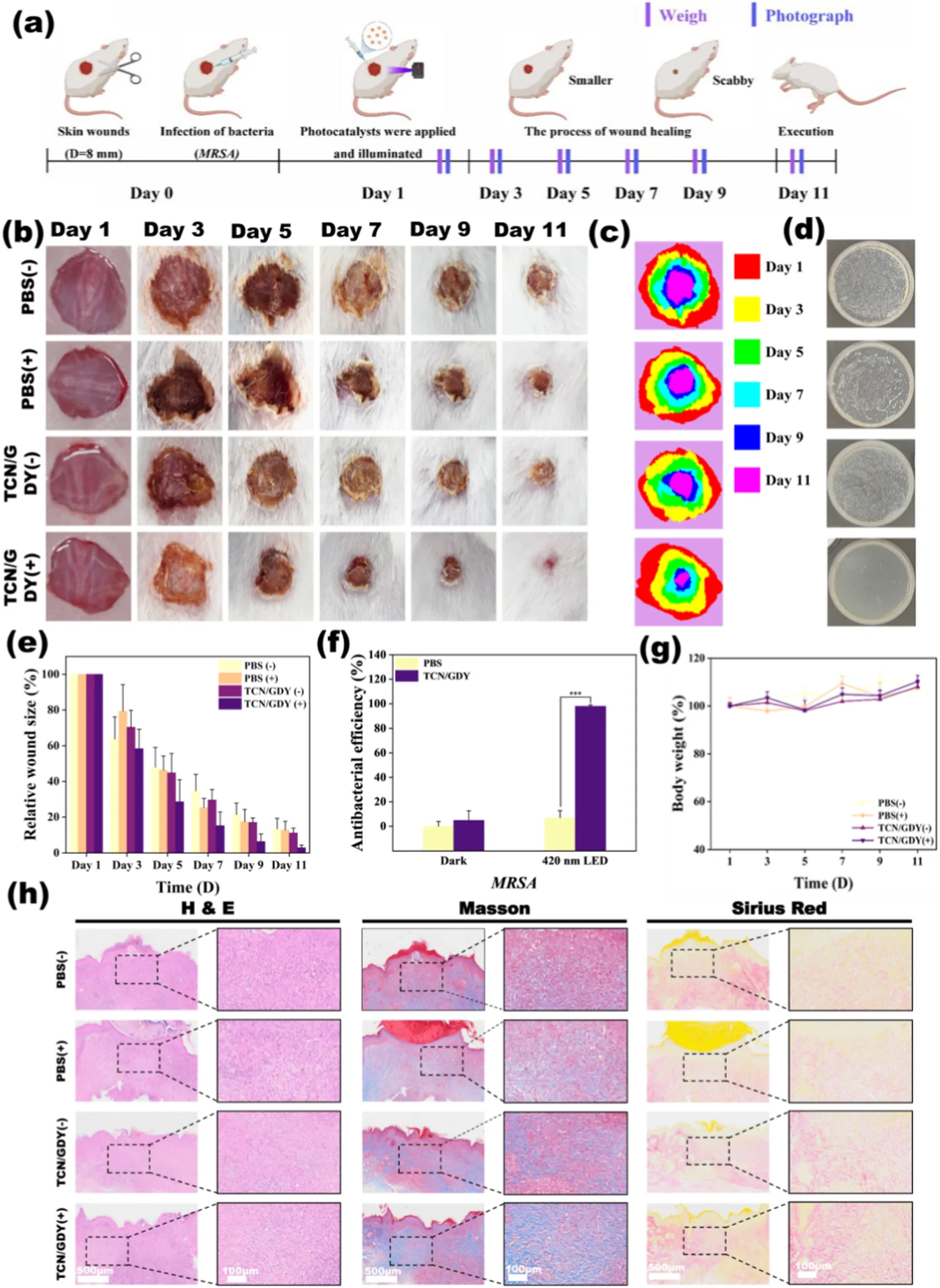
*In vivo* evaluation of TCN/GDY in an *MRSA*-infected wound model. (a) Schematic of wound creation, infection, treatment, and sampling. (b) Representative wound photographs at the indicated time points. (c) Visual summary of wound healing progression. (d) Representative *MRSA* colony plates recovered from infected wound tissue on day 11. (e) Quantitative wound closure rates. (f) Quantified antibacterial efficiency based on wound CFU counts (n = 5, ***P < 0.001). (g) Body weight changes during treatment. (h) Representative H&E, Masson, and Sirius Red stained wound sections collected on day 11. Data are presented as mean ± SD (n = 5, *p < 0.05, **p < 0.01, ***p < 0.001).

Bacterial recovery from wound tissue confirmed strong *in vivo* antibacterial activity (Fig. 9d–f). The TCN/GDY (+) group achieved a bacterial reduction efficiency of 97.98 ± 1.04%, consistent with the *in vitro* antibacterial trend. On day 11, wound healing was assessed histologically by H&E, Masson, and Sirius Red staining. The PBS (−) group exhibited incomplete epithelial regeneration with prominent scab formation, whereas the TCN/GDY (+) group displayed dense, well-organized epithelial tissue without scabs (Fig. 9h), indicating that TCN/GDY bio-heterojunction significantly accelerates wound closure compared to untreated infected wounds. To avoid the photothermal effects contributing to antibacterial properties, we also conducted temperature measurements. No significant temperature increase was observed (Fig. S21), indicating that photothermal effects did not contribute to the antibacterial activity. Inflammatory cell infiltration was markedly higher in the PBS (−), PBS (+), and TCN/GDY (−) groups relative to TCN/GDY (+) group (Fig. S22), suggesting that persistent *MRSA* infection maintained a pro-inflammatory microenvironment, delaying repair, while ROS generated by TCN/GDY bio-heterojunction under light rapidly controlled infection and reduced inflammation. Masson and Sirius Red staining further revealed enhanced collagen deposition in the TCN/GDY (+) group, with larger areas of mature collagen (blue in Masson, red in Sirius) compared with other groups (Figs. S23 and S24). The increased collagen content supports robust dermal regeneration. Although histological analysis showed no obvious tissue damage or abnormal inflammation in the TCN/GDY(+) group, ROS may also influence host cell signaling and repair, whereas excessive ROS can damage host tissues. The present study does not separate these direct host effects from the benefits of infection control or establish a ROS dose response relationship. Thus, future studies ought to measure oxidative stress markers in the wound tissues.

### 2.9 Biosafety evaluation

Short-term cellular responses were assessed using L929 fibroblasts and HUVECs. L929 cells showed preserved CCK-8 responses after 24 h exposure to T, TCN, and TCN/GDY and across the tested TCN/GDY concentrations at 24 and 72 h (Figs. S25–S26). In HUVECs, the CCK-8 absorbance at 450 nm after 24 h did not show a reduced signal for TCN/GDY relative to PBS (Fig. S27). Representative fluorescence images show predominantly green stained cells across the groups and provide qualitative evidence of short term cytocompatibility (Fig. S28). HUVEC scratch assay images at 0 and 12 h show greater gap closure in the TCN/GDY group under the tested conditions (Fig. S29). Hemolysis measurements provide additional short-term compatibility observations (Figs. S30–S31).

Routine hematology and representative H&E-stained sections of the heart, liver, spleen, lung, and kidney provide a short-term assessment during the 11-day wound study (Figs. S32 and S33). No significant adverse changes were detected in erythrocyte-related or leukocyte-related indicators apart from a decrease in neutrophil proportion that was consistent with alleviated infection-associated inflammation. H&E staining of the heart, liver, spleen, lung, and kidney showed no obvious tissue damage. These data support acceptable short term biosafety for the graphdiyne enabled TiO_2_/g-C_3_N_4_-based bio-heterojunction under the tested treatment conditions.

## 3 Conclusion

In this study, a graphdiyne enabled TiO_2_/g-C_3_N_4_ based bio-heterojunction was engineered by growing GDY on a TiO_2_/g-C_3_N_4_ scaffold. The calculations support contact polarization and interfacial electronic coupling, while photoelectrochemical and EPR measurements show an enhanced photoresponse and radical generation. A dual S-scheme like mechanism is consistent with these complementary observations. The L929 and HUVEC assays, hemolysis measurements, and short term *in vivo* observations support compatibility under the tested conditions. *In vitro* antibacterial experiments demonstrated that the visible light responsive TCN/GDY exhibits excellent antimicrobial performance against *S. aureus*, *E. coli* and *MRSA*. Multi-omics analysis identified treatment-associated changes in central metabolism, environmental sensing, and nutrient transport. In the superficial *MRSA*-infected open-wound model, TCN/GDY treatment was associated with lower bacterial burden and improved wound closure. These findings position the graphdiyne enabled TiO_2_/g-C_3_N_4_ based bio-heterojunction as a visible light antibacterial photocatalytic platform for infected wound healing.

## 4 Materials and methods

### 4.1 Materials

Urea, cuprous chloride, tetrabutyl titanate, acetic acid, methanal, barium sulfate, agar and methyl alcohol were purchased from Aladdin Co., Ltd. (Shanghai, China). HEB-TMS was obtained from Bichen Biochemical Technology Co., Ltd. (Shanghai, China). BCA protein concentration determination kit, and CCK-8 kit were obtained from Beyotime Biotechnology (Shanghai, China). 4% tissue cell fixative and Crystal Violet solution (0.1%) were bought from Solarbio Science & Technology Co., Ltd. (Beijing, China). α-MEM medium and Phosphoric acid buffer salt solution were purchased from BIOAGRIO Co., Ltd. Fetal calf serum was obtained from Tianhang Biotechnology Co., Ltd. (Zhejiang, China). Penicillin-streptomycin solution was purchased from Gibco Co., Ltd. Live/Dead staining kits were commercially obtained from Kemin Biotechnology Co., Ltd. (Shanghai, China).

### 4.2 Synthesis of g-C_3_N_4_

Three grams of urea were placed in a ceramic boat, wrapped and sealed with aluminum foil, and then annealed in a muffle furnace at 550°C for 2 h. After cooling to room temperature, the resulting yellow powder was collected and labeled as CN.

### 4.3 Synthesis of TiO_2_

The synthesis of TiO_2_ was adapted and modified from literature methods. First, 1 mL of tetrabutyl titanate was mixed with 30 mL of acetic acid and sonicated for 30 min. The resulting white suspension was transferred to a hydrothermal autoclave and reacted at 140°C for 12 h in an oven. After the reaction was complete and the system had cooled to room temperature, the white suspension was collected. The product was isolated by centrifugation (8000 rpm) and washed repeatedly with deionized water and anhydrous ethanol until the solution was nearly neutral. Finally, the obtained white powder was dried at 80°C for 12 h.

### 4.4 Synthesis of TiO_2_/g-C_3_N_4_

First, 0.5 g of TiO_2_ and 0.5 g of CN were added to an ethanol solution and sonicated for 30 min, followed by stirring for an additional 30 min. The mixture was then evaporated to dryness at 80°C. The resulting yellow powder was annealed in a muffle furnace at 550°C for 2 h to obtain the TiO_2_/g-C_3_N_4_ photocatalyst [29], labeled as TCN. For comparison, pure TiO_2_ annealed without the addition of g-C_3_N_4_ was prepared and labeled as T.

### 4.5 Synthesis of TiO_2_/g-C_3_N_4_/GDY

A deprotection-free growth method was employed to integrate GDY onto the surface of the TiO_2_/g-C_3_N_4_ photocatalyst. First, 100 mg of TCN powder was dispersed in a glass vial containing 20 mL of DMF. Subsequently, CuCl (10 mg) and 1 wt% HEB-TMS were sequentially added, and the mixture was sonicated for 30 min [30]. The sealed vial was then placed in an oven at 65°C and maintained for 24 h. After the reaction, the photocatalyst was washed several times with fresh DMF, anhydrous ethanol, and deionized water. Finally, the brown powder was dried at 60°C for 12 h and labeled as TCN/GDY.

### 4.6 Characterization and photoelectric testing

The morphological and structural characteristics were analyzed using transmission electron microscopy (TEM, JEM 2100 F), field emission scanning electron microscopy (FESEM, JSM-7800F) and X-ray diffraction (XRD, Shimadzu 7000). The elemental composition and chemical states were examined using X-ray photoelectron spectroscopy (XPS, Thermo Fisher ESCALAB 250Xi). The specific surface area and pore structure were examined through the N2 adsorption-desorption isotherms (BET, BELSORP MaxII). UV–vis diffuse reflectance spectra were recorded using an Agilent Cary 5000 UV–vis–NIR spectrometer. The photoluminescence spectra were acquired using the Hitachi F-7000 spectrophotometer. Radical trapping experiments utilized electron paramagnetic resonance spectroscopy (EPR, Bruker EMXplus). Transient photocurrent (I-t) and electrochemical impedance spectroscopy (EIS) were measured by an AUTOLAB electrochemical workstation. The conventional three-electrode configuration consisted of a platinum sheet serving as the counter electrode, an Ag/AgCl electrode functioning as the reference electrode, and an FTO conductive glass incorporated with the sample as the working electrode. The 0.5 M Na2SO4 solution was utilized as the electrolyte. A Xe lamp with a nominal power of 500 W was used as the illumination source, with the temperature of the light source regulated to approximately 15°C through a circulating water-cooling system.

Fourier-transform infrared (FTIR) spectra of T, TCN, and TCN/GDY were recorded using a Spectrum Two FTIR spectrometer (PerkinElmer, USA) over 4000–400 cm^−1^. Measurements were performed in transmission mode without KBr pellet preparation. Content of Cu was measured by ICP-OES in three separately prepared samples: an acid-treated TCN/GDY sample solution (1 mg/L), an aqueous extract collected after 24 h and an aqueous extract with xenon light irradiation collected after 24 h.

### 4.7 Bacterial culture and *in vitro* antibacterial evaluation

Gram-positive *Staphylococcus aureus*, Gram-negative *Escherichia coli*, and the drug-resistant strain *MRSA* were selected as representative bacteria. All strains were inoculated in Luria-Bertani (LB) medium and cultured in a shaking incubator at 37°C and 120 rpm. Subsequently, the *in vitro* antibacterial activity of TiO_2_/g-C_3_N_4_/GDY was evaluated. Briefly, bacterial suspensions at a concentration of 1 × 10^6^ CFU/mL were added to 24-well plates and incubated with different sample groups (500 μg/mL). After 30 min of exposure to a 420 nm LED or a Xe lamp, the bacteria were spread onto solid agar plates and incubated at 37°C for 24 h. Colonies were photographed and counted.

Both the biological assays and photoelectrochemical measurements used a Xe lamp with a nominal power of 500 W. The reported source-to-sample distance for biological illumination was 15 cm, with illumination covering the full sample area. Dark material and PBS controls were included in the antibacterial comparison. For the 420 nm LED condition, temperatures were compared at 0 and 30 min (Fig. S21).

### 4.8 *In vitro* anti-biofilm evaluation

A 1 mL suspension of methicillin-resistant *Staphylococcus aureus* (*MRSA*, 1 × 10^8^ CFU/mL) was added to 24-well plates and incubated at 37°C for 48 h. Free-floating bacteria were then removed by washing with PBS to obtain bacterial biofilms. The biofilms were co-cultured with different sample groups for 1 h. For the light-treated groups, the plates were exposed to LED irradiation for 30 min, followed by an additional 1 h of incubation. After treatment, the biofilms were washed with PBS before crystal violet staining. A 0.1% crystal violet solution (500 μL) was added and allowed to stain for 30 min. Excess crystal violet was removed by washing with PBS, and the stained biofilms in the wells were photographed for documentation. Finally, 500 μL of ethanol was used to dissolve the crystal violet, and the optical density at 600 nm was measured using a microplate reader. In the dark environment, biofilms were co-stained with PI and SYTO9 for 20 min, followed by three washes with PBS. Fluorescence images were acquired using confocal laser scanning microscopy.

### 4.9 Cytotoxicity evaluation

The biocompatibility of the photocatalysts with L929 cells was evaluated using the CCK-8 assay. L929 cells (100 μL, 2 × 10^4^ cells/mL) were seeded into 96-well plates and cultured for 24 h. The original medium was then replaced with fresh medium containing the photocatalysts. The cells were further cultured at 37°C under 5% CO_2_ for 1 or 3 days. Finally, the wells were washed three times with PBS, and 10 μL of CCK-8 solution along with 100 μL of fresh culture medium were added to each well. After incubating at 37°C for 1 h, the absorbance at 450 nm was measured to assess cell viability.

HUVECs were assessed after 24 h exposure to PBS, T, TCN, or TCN/GDY using CCK-8 measurements and live/dead fluorescence imaging. Representative HUVEC scratch assay images were recorded at 0 and 12 h for the same treatment groups.

### 4.10 Wound healing evaluation

*In vivo* animal experiments were conducted using 6-week-old female BALB/c mice in good health condition. The mice were divided into four groups: PBS (−), PBS (+), TCN/GDY (−), and TCN/GDY (+) (where “−” indicates no light exposure and “+” indicates light exposure), with five parallel samples per group. After gas anesthesia, circular wounds approximately 8 mm in diameter were created on the backs of the mice. To establish a wound infection model, 100 μL of pre-cultured *MRSA* (10^8^ CFU/mL) was applied to the wounds. On the following day, the respective treatments were applied directly to the exposed wound surface. For the illuminated groups, a 420 nm LED array positioned above the wound provided direct illumination for 30 min, without relying on transmission through intact overlying skin. Body weight was recorded every other day, and wound photographs were taken to document the healing process.

After 11 days of treatment, the mice were euthanized. Wound tissues were fixed with 10% formaldehyde and subjected to H&E staining, Masson staining and PSR staining. Blood samples were collected for hematological analysis. The animal ethics review application number is IACUC-20241223-06.

### 4.11 Theoretical calculations

First-principles calculations were performed using CP2K 2024.1 within the Gaussian and Plane-Wave framework, with PBE, GTH-PBE pseudopotentials, and SZV-MOLOPT-GTH basis sets. The plane-wave and relative cutoffs were 400 and 50 Ry, respectively, with a four-level multigrid and Γ-point sampling for the interfacial supercell. The SCF convergence threshold was 1.0 × 10^−6^ Hartree. The interface geometry was optimized before the frozen-fragment comparison. For contact analysis, the ternary structure and its frozen isolated TiO_2_, CN, and GDY components were evaluated with consistent simulation cells, atomic coordinates, and numerical settings; all four SCF calculations converged. The electron-number-density difference was defined as Δn = n_ternary_ − n_TiO2_ − n_CN_ − n_GDY_. Component electron-number changes were obtained using Mulliken, Hirshfeld, and real-space partitions of the same calculated contact redistribution. Real-space partition boundaries were additionally shifted by ±0.3 Å to examine the stability of the signs. Work functions were evaluated separately from the +z and −z vacuum levels as Φ = E_vac_ − E_F_. The contact-induced electron electrostatic potential-energy difference was referenced to its mean over z = 18–21 Å. With U = −eφ, its z derivative gives ΔE_z_ = (1/e)d(ΔU)/dz; field changes were averaged within ±0.3 Å of each interface centre. Additional definitions and numerical results are reported in the Supplementary Information.

### 4.12 Multi-omics analysis

Total RNA was extracted from individual *MRSA* samples using the cetyltrimethylammonium bromide (CTAB) method, followed by genomic DNA removal. RNA purity and concentration were initially evaluated using a NanoDrop2000 spectrophotometer (Thermo Fisher Scientific, USA). RNA integrity was further verified by agarose gel electrophoresis and confirmed with an Agilent Bioanalyzer 5300 system (Agilent Technologies, USA). High-quality RNA samples were subsequently used for library preparation and sequenced on the Illumina NovaSeq™ X Plus platform. All transcriptomic bioinformatic analyses were conducted using the Majorbio Cloud Platform (www.majorbio.com).

*MRSA* samples for metabolomic analysis were prepared as a separate sample set, with six biological replicates per group. Metabolites were extracted using 400 μL of 80% methanol containing 0.02 mg/mL L-2-chlorophenylalanine as an internal standard. Samples were homogenized in a cryogenic grinder (−10°C, 50 Hz, 6 min), sonicated at 5°C (40 kHz, 30 min), incubated at −20°C for 30 min, and centrifuged (13,000×g, 15 min, 4°C). The resulting supernatant was analyzed by LC–MS (Thermo Scientific Vanquish Horizon UHPLC-QExactive system) at Majorbio Bio-pharm Technology (Shanghai, China).

## CRediT authorship contribution statement

**Chongqing Yu:** Conceptualization, Writing – original draft. **Zujiang Liu:** Software, Investigation. **Jin Ye:** Resources, Software. **Ruixin Zhang:** Investigation. **Min Ren:** Investigation. **Fulin Deng:** Methodology. **Anning Zhao:** Visualization, Data curation. **Ali Abou-Hassan:** Data curation, Writing – review & editing. **Yichun Wang:** Methodology. **Yao Zhang:** Validation. **Valentim A.R. Barão:** Conceptualization. **Liqun Xu:** Funding acquisition. **Jie Xu:** Resources, Supervision. **Xi Rao:** Funding acquisition, Writing – review & editing, Supervision.

## Declaration of competing interest

The authors declare that they have no known competing financial interests or personal relationships that could have appeared to influence the work reported in this paper.

## Data Availability

Data will be made available on request.
