# Supplementary material for: A graphdiyne-enabled TiO2/g-C3N4 bio-heterojunction for visible-light antibacterial photocatalysis and infected wound healing
Source: Mater Today Bio. 2026 Oct 20;41:103765. doi: 10.1016/j.mtbio.2026.103765 (PMC13733462; doi:10.1016/j.mtbio.2026.103765)
Supplement: Supplementary file 1 [file mmc1.docx]

**A Graphdiyne‑Enabled TiO_2_/g‑C_3_N_4_ Bio‑Heterojunction for Visible‑Light Antibacterial Photocatalysis and Infected Wound Healing**

Chongqing Yu^1,^ ^2#^, Zujiang Liu^1#^, Jin Ye^1#^, Ruixin Zhang^1^, Min Ren^1^, Fulin Deng^1^, Anning Zhao^1^, Ali Abou-Hassan^3, 4^, Yichun Wang^2^, Yao Zhang^2^, Valentim A R Barão^5^, Liqun Xu^1*^, Jie Xu^2*^, Xi Rao^1*^

^1^BRICS Joint Laboratory on Biomedical Materials, School of Materials and Energy, Southwest University, Chongqing, 400715, PR China

^2^College of Stomatology, Chongqing Medical University; Chongqing Key Laboratory of Oral Diseases; Chongqing Municipal Key Laboratory of Oral Biomedical Engineering of Higher Education; Chongqing Municipal Health Commission Key Laboratory of Oral Biomedical Engineering, Chongqing 401147, China

^3^PHysicochimie des Électrolytes et Nanosystèmes InterfaciauX (PHENIX), Sorbonne Université, CNRS, 75005 Paris, France

^4^Institut Universitaire de France (IUF), 75231 Paris, France

^5^Department of Prosthodontics and Periodontology, Piracicaba Dental School, University of Campinas (UNICAMP), Piracicaba, São Paulo, Brazil.

^#^These authors contributed equally to this work.

^*^Corresponding author:

Xi Rao, Professor, E-mail: raoxiemail@swu.edu.cn

Jie Xu, Associate Researcher, E-mail: [501117@hospital.cqmu.edu.cn](mailto:501117@hospital.cqmu.edu.cn)

Liqun Xu, Professor, E-mail: [xulq@swu.edu.cn](mailto:xulq@swu.edu.cn)

Figure S1. Raman spectra of T and TCN.

Figure S2. FTIR transmission spectra of TiO_2_ (T), TiO_2_/g-C_3_N_4_ (TCN), and TiO_2_/g-C_3_N_4_/GDY (TCN/GDY).

Figure S3. XPS survey spectra of T, TCN, and TCN/GDY.

**Supplementary analysis of contact polarization and illumination dependent XPS**

**Ground state contact analysis**

The ternary stack and its frozen isolated components were evaluated using CP2K 2024.1 with PBE, SZV-MOLOPT-GTH basis sets, and 400/50 Ry cutoffs. The same simulation cell, fragment coordinates, and numerical settings were used for the contact comparison, and all four SCF calculations converged. The electron-number-density difference is Δn = n_ternary_ − n_TiO₂_ − n_CN_ − n_GDY_. Mulliken, Hirshfeld, and real-space partitions provide alternative descriptions of this same calculation, rather than independent replicate measurements.

Figure S4. Charge redistribution on contact in the idealized TiO₂/CN/GDY stack. (a) Planar electron-number-density difference between the ternary structure and its frozen isolated components. Positive and negative values denote accumulation and depletion, respectively; dotted lines mark the integration boundaries. (b) Component electron-number changes from three partitions. Positive values denote electron gain.

Table S1. Electron-number changes per simulated supercell relative to the frozen isolated components. Positive values denote electron gain. The three partitions analyse the same calculated contact redistribution.

| **Component** | **Mulliken ΔN** | **Hirshfeld ΔN** | **Real-space ΔN** |
| --- | --- | --- | --- |
| TiO₂ | +1.166 | +2.629 | +1.305 |
| CN | -2.569 | -4.045 | -3.122 |
| GDY | +1.403 | +1.441 | +1.816 |

Figure S5. Local density of states (LDOS) for the GDY layers.

Figure S6. Work functions and contact-induced electrostatic response. (a) Work functions of the isolated components and ternary stack referenced to the +z and −z vacuum sides. (b) Electron electrostatic potential-energy difference, ΔU = U_ternary_ − U_TiO₂_ − U_CN_ − U_GDY_, referenced to its mean over z = 18–21 Å. The fragment geometries and numerical settings are the same as those used for Figure S4.

Figure S7. Illumination-dependent XPS shifts calculated from the labelled peak positions in Figure 5. (a) All 12 components, identified by spectral region and dark-state binding energy. (b) Changes in peak separation relative to C1, located at 284.46 eV in the dark. The labels provide no uncertainty estimates, so no error bars or statistical significance are assigned.

Table S2. Binding-energy shifts calculated from the reported dark and illuminated peak positions. Each row represents the corresponding labelled component; ΔBE = BE_light_ − BE_dark_.

| **Region** | **Dark BE (eV)** | **Light BE (eV)** | **ΔBE (eV)** |
| --- | --- | --- | --- |
| C 1s | 284.46 | 284.14 | -0.32 |
| C 1s | 285.04 | 284.78 | -0.26 |
| C 1s | 286.46 | 285.93 | -0.53 |
| C 1s | 288.96 | 288.52 | -0.44 |
| N 1s | 398.48 | 398.63 | +0.15 |
| N 1s | 399.86 | 399.98 | +0.12 |
| N 1s | 401.11 | 401.35 | +0.24 |
| Ti 2p | 456.77 | 456.94 | +0.17 |
| Ti 2p | 458.48 | 458.74 | +0.26 |
| Ti 2p | 464.24 | 464.39 | +0.15 |
| O 1s | 529.87 | 530.02 | +0.15 |
| O 1s | 531.89 | 531.37 | -0.52 |

Table S3. Peak separations relative to the same C1 component, whose dark-state position is 284.46 eV. The separation change is ΔBE(X) − ΔBE(C1), where X denotes the target component.

| **Peak pair** | **Dark separation (eV)** | **Light separation (eV)** | **Change (eV)** |
| --- | --- | --- | --- |
| Ti main − C1 | 174.02 | 174.60 | +0.58 |
| N main − C1 | 114.02 | 114.49 | +0.47 |
| O main − C1 | 245.41 | 245.88 | +0.47 |

Figure S8. Time-dependent photocatalytic degradation profiles of formaldehyde over T, TCN, and TCN/GDY.

Figure S9. Pseudo-first-order kinetic fitting curves for photocatalytic formaldehyde degradation. Data are presented as mean ± SD (n = 3).

Figure S10. Apparent photocatalytic rate constants of the prepared samples. Data are presented as mean ± SD (n = 3).

Figure S11. Formaldehyde concentration during the extended irradiation and repeated-challenge experiment with TCN/GDY.

Figure S12. Representative colony images of *S.aureus* treated with PBS, T, TCN, and TCN/GDY under dark, 420 nm LED, or Xe-lamp irradiation.

Figure S13. Quantitative antibacterial efficiencies against *S. aureus*. Data are presented as mean ± SD (n = 3, **p* < 0.05, ***p* < 0.01, ****p* < 0.001).

Figure S14. Representative colony images of *E.coli* treated with PBS, T, TCN, and TCN/GDY under dark, 420 nm LED, or Xe-lamp irradiation.

Figure S15. Quantitative antibacterial efficiencies against *E.coli*. Data are presented as mean ± SD (n = 3, **p* < 0.05, ***p* < 0.01, ****p* < 0.001).

Figure S16. Cu concentrations measured by ICP-OES in an acid-treated TCN/GDY sample solution (Solution), a separately prepared aqueous extract collected after 24 h (24 h Extract(-)) and a separately prepared aqueous extract with xenon light irradiation collected after 24 h (24 h Extract(+)). Data are presented as mean ± SD (n = 3).

Figure S17. Global gene-expression profiles of samples from different treatment groups.

Figure S18. Principal component analysis (PCA) of samples from different treatment groups.

Figure S19. Volcano plot of differentially expressed genes (DEGs).

Figure S20. COG functional classification of differentially expressed genes.

Figure S21. Temperature measurements at 0 and 30 min of 420 nm LED irradiation. Data are presented as mean ± SD (n = 3; ns, *p* > 0.05).

Figure S22. Quantitative inflammation scores derived from H&E stained wound sections. Data are presented as mean ± SD (n = 5, **p* < 0.05, ***p* < 0.01, ****p* < 0.001, *****p* < 0.001).

Figure S23. Quantification of collagen deposition based on Masson stained wound sections. Data are presented as mean ± SD (n = 5, **p* < 0.05, ***p* < 0.01, ****p* < 0.001, *****p* < 0.001).

Figure S24. Quantification of collagen deposition based on Sirius Red stained wound sections. Data are presented as mean ± SD (n = 5, **p* < 0.05, ***p* < 0.01, ****p* < 0.001, *****p* < 0.001).

Figure S25. Viability of L929 fibroblasts after 24 h exposure to T, TCN, and TCN/GDY. Data are presented as mean ± SD (n = 3, **p* < 0.05, ***p* < 0.01, ****p* < 0.001).

Figure S26. Concentration-dependent viability of L929 fibroblasts exposed to TCN/GDY for 24 and 72 h. Data are presented as mean ± SD (n = 3, ns *p* > 0.05, **p* < 0.05, ***p* < 0.01, ****p* < 0.001).

Figure S27. CCK-8 absorbance at 450 nm for HUVECs after 24 h exposure to PBS, T, TCN, and TCN/GDY. Data are presented as mean ± SD (n = 3; ns *p* > 0.05; ***p* < 0.01).

Figure S28. Representative live/dead fluorescence images of HUVECs after 24 h exposure to PBS, T, TCN, and TCN/GDY.

Figure S29. Representative HUVEC scratch-assay images at 0 and 12 h after treatment with PBS, T, TCN, or TCN/GDY.

Figure S30. Representative photographs from the hemolysis assay of the TCN/GDY bio-heterojunction after incubation with erythrocytes.

Figure S31. Quantitative hemolysis rates of TCN/GDY after incubation with erythrocytes. Data are presented as mean ± SD (n = 3; ns *p* > 0.05).

Figure S32. Hematological parameters of mice in different treatment groups: (a) WBC, (b) RBC, (c) HGB, (d) HCT, (e) MCV, (f) MCH, (g) MCHC, (h) PLT, (i) MPV, and (j) RDW. Data are presented as mean ± SD (n = 5).

Figure S33. Representative H&E-stained sections of major organs (heart, liver, spleen, lung, and kidney) after different treatments.
